# Assessing the quality of record keeping for cesarean deliveries: results from a multicenter retrospective record review in five low-income countries

**DOI:** 10.1186/1471-2393-14-139

**Published:** 2014-04-12

**Authors:** Evelyn Landry, Celia Pett, Renee Fiorentino, Joseph Ruminjo, Cristina Mattison

**Affiliations:** 1 EngenderHealth, 440 Ninth Ave., New York, NY 10001, USA; 2 SUNY Downstate Medical Center, 450 Clarkson Ave, Brooklyn, NY 11203, USA; 3 McMaster University, 1280 Main St. West, CRL-209, Hamilton, Ontario, Canada

**Keywords:** Cesarean section, Record keeping, Partograph, Monitoring

## Abstract

**Background:**

Reliable, timely information is the foundation of decision making for functioning health systems; the quality of decision making rests on quality data. Routine monitoring, reporting, and review of cesarean section (CS) indications, decision-to-delivery intervals, and partograph use are important elements of quality improvement for maternity services.

**Methods:**

In 2009 and 2010, a sample of CS records from calendar year 2008 was reviewed at nine facilities in Bangladesh, Guinea, Mali, Niger, and Uganda. Data from patient records and hospital registers were collected on key aspects of care such as timing of key events, indications, partograph use, maternal and fetal outcomes. Qualitative interviews were conducted with key informants at all study sites to provide contextual background about CS services and record keeping practices.

**Results:**

A total of 2,941 records were reviewed and 57 key informant interviews were conducted. Patient record-keeping systems were of varying quality across study sites: at five sites, more than 20% of records could not be located. Across all sites, patient files were missing key aspects of CS care: timing of key events (e.g., examination, decision to perform CS), administration of prophylactic antibiotics, maternal complications, and maternal and fetal outcomes. Rates of partograph use were low at six sites: 0 to 23.9% of patient files at these sites had a completed partograph on file, and among those found, 2.1% to 65.1% were completed correctly. Information on fetal outcomes was missing in up to 40% of patient files.

**Conclusions:**

Deficits in the quality of CS patient records across a broad range of health facilities in low-resource settings in four sub-Saharan Africa countries and Bangladesh indicate an urgent need to improve record keeping.

## Background

Safe and timely access to cesarean section (CS) saves the lives of women experiencing serious obstetric complications. Yet, evidence suggests that the risks of short-term severe adverse maternal and perinatal outcomes are increased when CS is performed without medical indications [1-3]. CS also exposes women to an increased risk of complications and perinatal mortality in subsequent pregnancies [1,4,5]. The maternal health community is moving toward a decision-making framework based on evidence to ensure the appropriate use and quality of CS [6,7].

Routine monitoring and review of CS data is an important aspect of clinical audit and an underutilized tool for quality improvement in low-resource settings. Proposed health facility indicators for monitoring the quality of CS include: indications; case fatality rates; stillbirth and early neonatal death rates; duration between the decision to perform CS and the procedure; administration of prophylactic antibiotics; and use of the partograph [6]. Such indicators need to be validated and operationalized as part of quality improvement efforts, to detect where missed opportunities and/or substandard care can lead to disaster. Clinical audit of CS can help to ensure that CS is being performed for valid clinical reasons and reinforce its appropriate use: reducing the number of CSs performed unnecessarily, and/or highlighting the need to increase access in settings where women are dying for lack of CS. Provider performance can be improved with quality data from clinical audits, ongoing feedback, coaching, and support [8].

Between 2007 and 2013, EngenderHealth’s Fistula Care project provided technical assistance to strengthen prevention and treatment services for fistula, which included CS services, in 10 countries. This retrospective record review study was undertaken in five countries to identify areas for improvement by reviewing key details about CSs from patient records, such as use of the partograph, and to determine if there were challenges to recording and reporting CS data which could be strengthened. These findings identified priority areas for Fistula Care to focus its technical assistance to improve quality of care for CS.

## Methods

### Study sites

Nine facilities in five countries (Bangladesh, Guinea, Mali, Niger, and Uganda) were selected to participate in this record review study, based on their willingness and interest to address quality improvement for CS. National institutional delivery rates in these countries vary from around 30% in Bangladesh [9] and Niger [10], to 40% in Guinea [11], and between 50-60% in Mali [12] and Uganda [13]. National CS rates in 2008 were all below 5% [14] except notably in Bangladesh where rates nearly doubled from 9% to 17% over the five-year period 2007-2011 [9].

Data collection was carried out in 2009 and 2010. The study sites included six urban government facilities in Guinea (n = 2), Mali (n = 1), and Niger (n = 3); two rural faith-based facilities in Uganda; and one rural private hospital in Bangladesh. All facilities are referral centers, offering round-the-clock emergency obstetric and newborn care and serving large urban or rural catchment areas.

The study protocol was reviewed and approved by EngenderHealth following the agency’s research standard operating procedures and by the funding agency, United States Agency for International Development (USAID). Each participating hospital’s ethical review committee approved the study protocol and consented to participate in the record review. Individual consent was obtained for key informant interviews.

### Study sample

The record review sample consisted of 350 CSs (emergency or nonemergency) from calendar year 2008 from each study facility. A random sample was drawn from the facility’s operating room register (using a random number table); at sites where fewer than 350 CSs were performed in 2008, all were reviewed. One facility performed 376 CSs in 2008; all cases were reviewed there.

Key informant interviews (with 3–9 persons per facility) were conducted by the research teams to elicit qualitative descriptions of context and challenges from those involved in providing CS care (e.g., obstetrician/gynecologists, nurses, and midwives) and management of records and/or reporting (e.g., record room staff and nurses).

### Data collection tools

Data for the study were collected using a patient record abstraction form and key informant interview guides. We adapted the CS record abstraction form from the Averting Maternal Death and Disabilities (AMDD) project’s needs assessment tool for emergency obstetric services [15].

Two-person consultant teams from each country were trained to administer the tools: one physician familiar with obstetrics and the medical record-keeping systems in that country (designated as the lead consultant), and one research assistant. The physician was responsible for extracting clinical information from the clinical files.

The record abstraction tool included patient profile variables, current delivery referral history, and history of previous CS. Cesareans were classified as emergency or elective. When this information was not recorded in the patient file, the procedure was coded as an emergency if the decision was made after the woman had started active labor and as elective if the decision was made before active labor started. Other variables included timing of key events (e.g., admission and decision to perform CS); use of the partograph; primary indication; and maternal and fetal outcomes.

The lead consultant assessed partograph quality by using a nine-point checklist developed by Fistula Care. The partograph was assessed as completed correctly if the responses to all nine questions on the checklist were yes: 1) first cervical dilatation charted correctly on alert line; 2) cervical dilation plotted at least every 4 hours; 3) descent of presenting part checked and recorded during labor; 4) contractions assessed and recorded at least half hourly when in active labor; 5) state of membranes assessed and if ruptured, color of liquor recorded; 6) fetal heart rate recorded at least half hourly during labor; 7) mother’s blood pressure checked and recorded at time of admission and during labor; 8) mother’s pulse checked and recorded at admission and during labor; and 9) documentation of augmentation or other medication in labor.

The consultant was instructed to determine if the action line on the partograph had been reached or crossed while plotting cervical dilation and to record the number of hours on the data collection tool if the action had been crossed

The list of potential CS indications was expanded from the AMDD list of precoded indications to include a wider range of indications based on other published research [16-19]. Data collectors recorded the indication exactly as it was listed in the patient file and recorded verbatim other indications not listed in the tool. During our analysis, for simplicity, some indications were merged and recoded as a single indication (e.g., prolonged labor and failure to progress in labor were merged) (Table 1). Some of the “other” recorded indications (which were not part of the precoded options) found in patient records were recoded to indications on our final list (e.g., retracted/contracted pelvis and big baby to obstructed labor; cervical dystocia to failure to progress in labor/prolonged labor; arm prolapse/presentation to malpresentation; cardiopathy, cerebral malaria, and HIV to maternal medical disease). Data that did not include enough information to enable a clear determination about the indication were coded as “other/not enough information” in our analysis.

**Table 1:** Expansion of CS indications from AMDD tool to Fistula Care tool and final recoding

**CS indications, recoded for final analysis**	**Fistula Care data collection tool**	**AMDD CS record review tool**
**Maternal indications**		
Obstructed labor (including failed trial of labor, deformed pelvis)	• Obstructed labor	
	• Failed trial of labor	
	• Deformed pelvis	
Uterine rupture	• Uterine rupture	
Major antepartum hemorrhage & placenta previa (grade 3 or 4)	• Major antepartum hemorrhage & placenta previa (grade 3 or 4)	Placenta previa
Cephalopelvic disproportion (CPD)	• Cephalopelvic disproportion (CPD)	CPD (listed with prolonged labor)
Failure to progress in labor, including prolonged labor	• Failure to progress in labor	Prolonged labor (listed with CPD)
	• Prolonged labor	
Failed induction	• Failed induction	Failed induction
Previous CS	• Previous CS	Previous scar
	• Uterine scar from other previous surgery	
Genitourinary fistula or third-degree tear repair	• Vesico-vaginal fistula postrepair	Vesico-vaginal fistula
	• Vesico-vaginal fistula	
Antepartum hemorrhage (excluding those for absolute indications and including abruptio placentae)	• Antepartum hemorrhage (excluding those for absolute indications and including abruptio placentae)	Placenta abruptio
Maternal medical diseases (e.g., sickle cell anemia, HIV)	• Maternal medical disease (e.g., sickle cell anemia, HIV)	
Severe preeclampsia or eclampsia	• Eclampsia/severe preeclampsia	Eclampsia/severe preeclampsia
Psychosocial indications (including maternal request)	• Psychosocial/maternal/family request	Maternal distress
Precious pregnancy	• Precious baby	
**Fetal Indications**		
Fetal compromise (including fetal distress; cord prolapse/presentation; and severe intrauterine growth retardation)	• Fetal distress	Fetal distress
	• Cord prolapse/presentation	Cord prolapse
	• Severe intrauterine retardation	
Breech presentation	• Breech presentation	Breech with footling/ malpresentation
Multiple gestation	• Multiple gestation	Multiple gestation
Malpresentation (including transverse, oblique, and brow)	• Malpresentation (including transverse, oblique, and brow)	Breech with footling/ malpresentation
Other/not enough information	• Other^	Other

^Other indications include those found in patient records which were not part of the pre-coded options but were recoded to indications on our final list (e.g. big baby to obstructed labor. Data that did not include enough information to enable a clear determination about the indication were coded as “other/not enough information”.

When individual patient files could not be located, hospital registers (e.g., from the delivery room, operating theater, referral, and maternity ward) were used to locate data of interest. The tool was translated into French for use at West African facilities (see Additional file 1).

### Data analysis

The information from the key informant interviews from Bangladesh and Uganda was collated and summarized by the lead research consultants; the information from the other countries was summarized by one of the authors. Data cleaning and analysis of the CS data were performed using the statistical software package SPSS 20.0. Results are presented by study site. In some instances, data were missing from the patient files; we have noted variables for which more than 10% of data were missing. For confidentiality purposes, site names are concealed and are designated by country name and a letter, if there were multiple sites in a country (e.g., Guinea A, Guinea B).

No statistical tests were conducted by study site, as we never intended to compare practices across sites. The descriptive findings from the record reviews were shared with key stakeholders at each facility and served as baseline assessments. Individual in-depth reports were prepared for each study site; these reports included recommendations and actions to improve quality of services and record keeping (K. Beattie, personal communication, April 5, 2013).

## Results

### Profile of study sites

A total of 57 key informants were interviewed and 2,941 records reviewed. The 2008 institutional CS rates at the study sites ranged from 7% to 53% (Table 2). While all sites served as referral centers, their size (the number of maternity beds/total beds) and the number of annual deliveries varied greatly across sites. Obstetrician/gynecologists, general surgeons, or general practitioners performed CS at all sites except in Guinea, where at the time of the study only general surgeons performed CS. All sites reported using paper-based systems for client records and multiple logbooks/registers for tracking patient information in maternity wards, many of which were duplicative, with missing data. Many maternity ward and record room personnel reported that staff often did not understand the importance of proper record keeping and lacked training or motivation. Other record-keeping challenges included lack of data management guidelines and standards, poor filing systems, lack of space for storing medical records, and infrequent data review meetings. Most patient medical records reviewed had few standardized variables for documenting care.

**Table 2:** Profile of study sites, 2008

	**Bangladesh**	**Guinea**		**Mali**		**Niger**		**Uganda**	
		**A**	**B**		**A**	**B**	**C**	**A**	**B**
Type of institution	Private	Public	Public	Public	Public	Public	Public	Faith-based	Faith-based
Location	Rural	Urban	Urban	Urban	Urban	Urban	Urban	Rural	Rural
Total no. of maternity beds/total no. of hospital beds	80/750	20/105	30/119	24/128	36/186	36/382	2/53	34/266	50/200
Providers who perform CS									
*Obstetrician-gynecologists*	X	-	-	X	X	X	X	X	X
*General surgeons or general practitioners*	-	X	X	X	X	X	X	X	-
No. of deliveries in 2008	2,178	1,136	719	1,048	1,868	1,375	4925	2,929	1,778
No. of CS deliveries in 2008	1,068	277	379	269	302	688	324	998	663
2008 institutional CS rate	49%	24%	53%	26%	16%	49%	7%	34%	37%
No. of CS deliveries reviewed	350	277	376	269	299	349	324	348	349
% of patient files found	100.0%^1^	92.1%	61.2%	36.8%	98.7%	65.0%	67.3%	78.4%	95.1%
Number of key informants from facility interviewed	8	7	8	8	4	4	3	6	9

^1^Includes 41 records (11.7%) that were partially found.

### Characteristics of women

Sociodemographic characteristics (age, parity, place of residence) of women who had a CS are shown by facility in Table 3. Patient records indicate that more than 50% of women who had a CS at the Mali and Niger sites had been referred for labor care from another facility; most of these women came with no accompanying documentation or partograph. Data were not collected about the stage of labor the woman was in when she arrived from the referring facility.

**Table 3:** Percentage distribution of characteristics of women undergoing CS, by study site

	**Bangladesh**	**Guinea A**	**Guinea B**	**Mali**	**Niger A**	**Niger B**	**Niger C**	**Uganda A**	**Uganda B**
	**n = 350**	**n = 277**	**n = 376**	**n = 269**	**n = 299**	**n = 349**	**n = 324**	**n = 348**	**n = 349**
Age 25 years or less	70.3	55.2	55.3	62.8	41.5	53.6	50.0	67.2	53.9
Primiparous^1^	57.0	35.2	33.7	45.9	28.0	35.9	33.0	34.9	37.4
Rural residence^2^	98.6	41.2	70.5	31.6	61.2	50.7	58.0	77.6	63.0
Previous CS	12.6	11.9	26.9	12.6	16.4	15.5	12.7	43.1	30.8
Referred to facility	0.9	12.6	38.6	51.3	66.9	88.3	56.2	10.6	22.6

^1^Data missing for more than 10% of cases reviewed at Guinea A.

^2^Data missing for more than 10% of cases reviewed at Uganda A.

### Use of the partograph

No partographs were found in patient files at both Guinea sites, and fewer than 2% of patient records at the Bangladesh site had partographs (Table 4). The majority of patient files from the Niger sites included a partograph; however, at two of these sites, fewer than 3% were completed correctly.

**Table 4:** Percentage distribution of partograph use, by study site

	**Bangladesh**	**Guinea A**	**Guinea B**	**Mali**	**Niger A**	**Niger B**	**Niger C**	**Uganda A**	**Uganda B**
	**n = 350**	**n = 277**	**n = 376**	**n = 269**	**n = 299**	**n = 349**	**n = 324**	**n = 348**	**n = 349**
Partograph used	1.4	0.0	0.0	23.8	97.3	96.8	99.4	23.9	18.3
Partograph completed correctly^1^	20.0	-	-	34.4	2.1	65.1	0.2	23.9	18.3
Partograph action line crossed	0.0	-	-	4.7	7.6	1.5	5.0	-	46.9

^1^The partograph was assessed as completed correctly if the responses to all nine questions on the checklist were yes: 1) first cervical dilatation charted correctly on alert line; 2) cervical dilation plotted at least every 4 hours; 3) descent of presenting part checked and recorded during labor; 4) contractions assessed and recorded at least half hourly when in active labor; 5) state of membranes assessed and if ruptured, color of liquor recorded; 6) fetal heart rate recorded at least half hourly during labor; 7) mother’s blood pressure checked and recorded at time of admission and during labor; 8) mother’s pulse checked and recorded at admission and during labor; 9) documentation of augmentation or other medication in labor.

### Type of CS and indications

Data were not available about the type (emergency or elective) of CS for more than a third of the cases at three sites (Bangladesh, Mali, Uganda A). Among the partographs reviewed, the percentage showing the action line had been crossed (indicating the need for an intervention, such as labor augmentation, or CS) ranged from 1.5% to 46.9% (Table 4).

At four of the nine sites, at least nine out of 10 CSs were identified as emergency in the patient file (96%, 99%, and 100% at Niger A, B, and C, and 94% at Guinea A), and at two other sites, more than three in four were classed as emergency (75% at Guinea B and 86% at Uganda B). Emergency interventions represented a smaller percentage of all CSs at Uganda A (60%) and in Mali (46%); just 30% of procedures in Bangladesh were considered emergencies.

Key informants from all study sites stated that there were no formally documented CS classification systems in place. Nearly all of the files reviewed included a recorded indication for CS. Maternal indications accounted for two-thirds or more of CSs at all sites except Bangladesh. The leading maternal indications were obstructed labor (including conditions that pose high risk for obstructed labor), followed by failure to progress/prolonged labor, uterine rupture, and previous CS (Table 5). Cervical dystocia was used to describe prolonged labor at six of the nine sites. Fetal indications ranged from 9.4% to 27.2%; the leading indication was “fetal compromise”; we did not collect additional information from the patient file, such as fetal heart rate, to validate the fetal compromise indication. In Bangladesh, one-third of indications were classified as “other, not enough information”; “postdates” accounted for the majority of the indications in this category (60%; n = 70).

**Table 5:** Percentage distribution of primary CS indication, by study site

**Primary indication**	**Bangladesh**	**Guinea A**	**Guinea B**	**Mali**	**Niger A**	**Niger B**	**Niger C**	**Uganda A**	**Uganda B**
	**n = 350**	**n = 277**	**n = 376**	**n = 269**	**n = 299**	**n = 349**	**n = 324**	**n = 348**	**n = 349**
**Maternal indications**	**41.5**	**89.5**	**87.0**	**72.4**	**69.1**	**76.7**	**70.7**	**73.3**	**78.2**
Obstructed labor (including failed trial of labor, deformed pelvis)	2.0	59.2	52.7	16.4	10.4	16.0	9.3	14.9	30.9
Failure to progress/prolonged labor	5.1	2.5	0.3	8.6	9.0	5.7	7.4	19.5	16.3
Uterine rupture	0.3	7.2	11.4	10.0	20.7	9.5	14.2	0.3	0.3
Previous CS	11.4	0.7	11.4	3.0	3.7	5.2	2.5	18.7	10.9
Severe preeclampsia or eclampsia	11.1	0.0	0.3	13.4	7.4	17.2	14.2	1.7	2.0
Cephalopelvic disproportion	5.4	6.1	4.8	11.2	6.7	4.6	5.2	11.5	5.7
Antepartum hemorrhage and grade 3 or 4 placenta previa	1.4	4.7	4.0	3.7	5.4	9.7	9.6	2.3	4.6
Antepartum hemorrhage, excluding absolute indications, including abruptio placentae	1.4	8.7	0.3	5.2	0.7	6.9	5.9	2.6	0.0
Precious pregnancy*	2.3	0.4	0.5	0.4	2.3	0.3	0.3	0.6	2.3
Genitourinary fistula or third-degree tear repair	0.0	0.0	1.3	0.4	2.0	0.3	1.2	0.0	2.0
Maternal medical disease^	0.3	0.0	0.0	0.0	0.3	0.3	0.6	1.1	1.4
Failed induction	0.3	0.0	0.0	0.0	0.0	0.0	0.3	0.0	1.4
Psychosocial, including maternal request	0.0	0.0	0.0	0.0	0.3	0.0	0.0	0.0	0.3
**Fetal indications**	**24.9**	**9.4**	**12.5**	**22.7**	**25.4**	**20.1**	**27.2**	**25.8**	**19.8**
Fetal compromise (fetal distress, including cord prolapse/presentation, severe intrauterine growth retardation)	18.3	5.1	7.2	12.3	10.0	6.0	16.4	14.9	7.2
Malpresentation (including transverse, oblique, brow)	2.0	4.3	4.8	4.8	12.4	10.9	9.6	5.5	10.6
Breech presentation	4.6	0.0	0.5	5.6	3.0	2.3	0.9	3.7	1.7
Multiple gestation	0.0	0.0	0.0	0.0	0.0	0.9	0.3	1.7	0.3
**Other/not enough information**	**33.1**	**1.1**	**0.5**	**4.8**	**5.4**	**2.9**	**2.2**	**0.9**	**2.0**
**No indication recorded**	**0.9**	**0.0**	**0.0**	**0.4**	**0.3**	**1.4**	**0.0**	**0.0**	**0.0**

*Precious pregnancy is defined as a pregnancy coming after a series of pregnancy losses, such as miscarriages or still births.

^Pre-existing conditions, such as cardiac disease or co-morbidities, such as HIV. While preexisting hypertension predisposes to preeclampsia/toxemia and eclampsia, these conditions are excluded from the category, because they are pregnancy-specific.

### Timing of care and prophylactic antibiotics

Data on the timing of key events were frequently missing across all sites (Table 6). Providers at most sites recorded time of admission and time of birth in patient records. However, data on other critical timing events, such as decision for surgery made and surgery start time, were rarely recorded; these data were missing for one or both variables in more than 80% of cases, making it impossible to assess the time interval between decision and incision.

**Table 6:** Percentage distribution of records found with information on the timing of key events and administration of prophylactic antibiotics, by study site

	**Bangladesh**	**Guinea A**	**Guinea B**	**Mali**	**Niger A**	**Niger B**	**Niger C**	**Uganda A**	**Uganda B**
	**n = 350**	**n = 277**	**n = 376**	**n = 269**	**n = 299**	**n = 349**	**n = 324**	**n = 348**	**n = 349**
Time of admission	82.9	19.5	1.6%	51.7	92.3	90.5	96.0	NA	50.4
Time of first examination	4.6	0.4	0.3	5.6	59.9	75.1	67.6	NA	59.0
Time decision was made to do CS	5.1	0.4	0.0	6.7	2.7	3.4	27.8	NA	50.9
Time of skin incision	0.0	0.0	66.5	34.2	2.3	0.0	1.9	0.0	0.0
Time of birth	99.1	72.9	67.0	75.5	96.3	91.7	98.8	NA	88.0
Records with surgical consent form	87.4	0.0	0.3	0.0	0.0	0.0	0.0	NA	92.8
Prophylactic antibiotics administered	88.3	97.8	98.6	35.7	97.0	63.6	67.0	85.3	85.7

NA = not available. The data collection form was modified to collect this information only after the study was completed at this site.

WHO’s best practice guidelines recommend the use of prophylactic antibiotics for all women undergoing a CS [20]. The records indicated that administration of prophylactic antibiotics ranged from 35.7% to nearly 100%. Data on this measure were missing from more than 10% of the files in Bangladesh, Mali, two of the Niger sites, and one of the Uganda sites.

### Maternal outcomes

Information about whether women experienced complications was missing in at least one-third of the records reviewed from five sites. Documented maternal complications ranged from 2.9% to 28.4%. The most commonly recorded complication across all sites was anemia (9.1–74.1%), followed by wound infection (4.3–62.2%) (data not shown).

Data on whether the woman died or survived the CS were missing for more than 10% of files at three sites; a total of 46 maternal deaths were found in the records reviewed. The percentage of recorded maternal deaths ranged from <1% to 3%; the largest percentages of deaths were from the three Niger sites and one site in Guinea (Table 7). The primary cause of death was recorded for only five cases. Partographs were found in 28 of the 46 cases.

**Table 7:** Percentage distribution of maternal and fetal outcomes, by study site

	**Bangladesh**	**Guinea A**	**Guinea B**	**Mali**	**Niger A**	**Niger B**	**Niger C**	**Uganda A**	**Uganda B**
	**n = 350**	**n = 277**	**n = 376**	**n = 269**	**n = 299**	**n = 349**	**n = 324**	**n = 348**	**n = 349**
**Maternal outcomes**									
Maternal death	0.0	0.4	3.2	0.7	3.3	2.6	2.8	0.0	0.9
No information on maternal outcome	11.4	0.7	2.5	0.4	0.3	5.4	25.0	23.9	6.0
**Fetal outcomes**									
Born alive	86.6	74.4	72.1	81.4	65.2	69.6	65.4	84.2	51.3
Perinatal Death	1.4	23.1	24.5	18.6	34.4	30.1	34.0	4.9	8.6
Stillbirth	*0.0*	*98.4*	*90.2*	*48.0*	*78.6*	*23.8*	*20.0*	*35.3*	*63.1*
Early Neonatal deaths	*0.0*	*0.0*	*8.7*	*16.0*	*6.8*	*17.1*	*3.6*	*52.9*	*36.7*
Missing information on timing of death	*100.0*	*1.6*	*1.1*	*36.0*	*14.6*	*59.0*	*76.4*	*11.8*	*0.0*
Missing information on birth outcome	12.0	2.5	3.5	0.0	0.3	0.3	0.6	10.9	40.1

Focusing on the four sites with the largest number of deaths (40 deaths in total from the three Niger sites and Guinea B), seven of the 40 deaths occurred in the intrapartum period and 28 in the postpartum period; data were missing for five cases. One death (Guinea) was recorded as being an elective CS. While the partograph was used in all cases of maternal death at the three Niger sites, it was only completed correctly for five cases at one site. It was not used at the Guinea site. All of the women from Niger sites A and B (n = 19) had been referred; half of the women at the other two sites had been referred. Five of the six women at the Guinea site came with referral notes; at the Niger sites, only two women arrived with notes. Fifteen of the women who died at two of the Niger sites and the Guinea site had a CS indication of uterine rupture; at Niger B, severe eclampsia/preeclampsia was listed for five of the nine deaths.

### Fetal outcomes

More than 10% of files at three sites included no information on birth outcomes (Table 7). The percentage of recorded perinatal deaths ranged from 1.4% to 34.4%. Data on timing of death were missing from all files in Bangladesh and from 1.1% to 76.4% at the other sites. Among records with information about the timing of death, stillbirths ranged from 23.8% to 98.4% and early neonatal deaths from 3.6% to 52.9%. In more than half of all cases of maternal death at all sites (except Niger B), the baby also died. The partograph was used in nearly all of the recorded perinatal deaths at the Niger sites; it was rarely used at any of the other sites. Among early neonatal deaths, the primary recorded cause of death was asphyxia and birth trauma (ranging from 9.1% to 33.3%) (data not shown).

## Discussion

### Improving the quality of record keeping

Individual patient files were missing for more than 20% of all CSs at five of the nine sites. For certain key quality of care indicators, such as partograph monitoring, time of decision to perform CS, prophylactic antibiotic administration, and maternal and fetal outcomes, 10% or more of the data were missing from more than three sites. However, CS indication data were found in nearly all of the cases reviewed. Most of the women who had been referred arrived without notes or a partograph. Incomplete, inaccurate, and inaccessible medical records have the potential to adversely impact decision making and care. Improved record keeping could facilitate routine monitoring, reporting, and clinical audits that might help facility staff identify deficiencies in care [21,22]. Findings from this study indicate a pressing need to improve record keeping across study sites and referring facilities.

The decision-to-delivery (DTD) interval was not recorded in most files, and more than one-third of files at three sites did not include information about whether the CS was emergency or elective. The time interval between the decision to do a CS and the intervention is critical in obstetric emergencies, particularly in low-resource settings, as delays in care are a significant contributor to maternal and newborn morbidity and mortality [23]. Existing DTD guidelines target high-resource settings and are unlikely to be feasible in low-resource settings [24,25]. To improve CS record keeping, we recommend the inclusion of the DTD interval in standardized patient records to better estimate the magnitude of delays and to establish attainable DTD standards in this context. Patient flow analyses can identify sources of delay, and obstetric “emergency drills” and case simulations can help prepare and motivate staff while improving performance [26,27].

Poor record keeping raises critical questions about the care provided: if there is no documentation, was care provided? Or did staff believe that the care provided was not significant enough to document? The quality of record keeping (and care) suffers when providers are overburdened and facilities are understaffed. Key informants acknowledged the need to train and motivate staff in the importance of recording keeping for improving quality of care. To be effective, training will need to be supported with ongoing facilitative supervision.

Documentation found in our study was often duplicative and lengthy. Computerization of patient records is a long-term goal for many facilities to improve quality and access to patient information [21,22]. In the meantime, improvements to paper-based systems can be achieved by developing a standardized individual patient maternity and CS record, including the partograph. A “tick box” format could be used to record information and care given, similar to WHO’s Safe Childbirth Checklist [27]. This would be easier and quicker to complete, avoid duplication, and act as a useful teaching job aid for providers, prompting them to perform essential elements of care while facilitating retrieval of data for routine review.

### Improving the quality of labor monitoring—use of the partograph

Our findings indicate that partograph use is disappointingly low. The high numbers of incomplete and incorrectly completed partographs suggest that many providers do not understand how to use it properly or are unable to do so because of workload demands. In 1.5% to 46.5% of cases, when the action line was crossed, partograph findings did not translate into action. It is possible that these were completed after the delivery, a practice that has been reported in the literature [28,29]. However, we cannot corroborate this from our data.

A recent Cochrane review of the effect of partograph use on clinical outcomes concludes that there is no evidence that it has any effect on intrapartum care. However, the review also stated that partograph use may be of some benefit in settings with poorer access to health care resources. Additionally, studies have shown that partograph use and early interventions for women experiencing a delay in the progress of labor have contributed to some reduction in CS rates [30].

In low-resource settings such as our study sites, we suggest the partograph remain an important (and often the only available) clinical decision-making tool for labor monitoring and management [31]. In particular, it is valuable for diagnosing prolonged and obstructed labor, leading indications for CS in our review. Further research to determine effective approaches for partograph training and implementation may be a valuable investment in improving the quality of labor monitoring and clinical decision making.

### Provision of care

Data on CS type and indications have the potential to reveal important information about the quality of procedures performed [6,19] and provide insights that are masked by institutional CS rates alone. The institutional CS rates for Bangladesh, Guinea B, and Niger B were relatively similar (49%, 53%, 49%, respectively) but had different indication profiles—at the Guinea and Niger sites, the leading indications were obstructed labor, uterine rupture, previous CS, and eclampsia/severe preeclampsia. In Bangladesh, one-third of the CS indications were listed as “other, not enough information”, suggesting that some CSs may not have been medically justified, potentially exposing women to greater risk of adverse outcomes.

None of the study sites employed a formal CS classification system. Clinicians at study sites used a wide range of terminology to describe CS indications. For example, conditions resulting in obstructed labor were described in a multitude of ways, including deformed or contracted pelvis, big baby, and failed trial of labor. The variety of overlapping terminology shown by our study echoes the multiple codes listed in the WHO International Classification of Disease for causes of prolonged and obstructed labor and draws attention to the need for agreement on a simplified and standardized global terminology to describe these common conditions [32]. Standardized terminology for CS indications would also facilitate clinical audit and monitoring of trends. Multiple classification systems have been proposed, based on clinical indications, “degree of urgency”, or patient characteristics, but none have been extensively implemented [33].

### Maternal outcomes

Data on postoperative maternal complications were missing from one-third or more of the records at five sites. While it may be possible that women did not experience any complications, given that many other variables were also missing from patient records, it is possible that this information was not recorded. Delivery by CS is major surgery, and one would expect to see complications, even minor ones (such as wound infection, adverse reactions to medications, or abnormal bleeding), to be recorded in patient files. In addition, data on whether the woman survived or died were missing in more than 10% of the files at three study sites.

The majority of the recorded maternal deaths occurred at four sites (n = 40); most of the women had been referred but without notes. These data suggest that the women experienced delays in reaching the referral center and/or after arrival. The large number of women in the study population who experienced uterine rupture is further evidence of severe delay in taking the necessary action. Overall, the maternal case fatality rates in this sample are high. We do not have data to ascertain how generalizable these rates are compared to other low resource settings. We strongly recommend increased investment in strengthening the capacity of peripheral facilities and referring providers to properly and efficiently diagnose, refer and transfer women to emergency obstetric care.

### Fetal outcomes

Perinatal outcomes (stillbirth and early newborn death) have been proposed as a facility indicator of CS quality of care [6]. In this study, data on birth outcomes were missing in 10% or more of the files at three sites. The majority of fetal deaths (48% or more) were classified as stillbirths at five sites; early neonatal deaths ranged from 3.6% to 52.9% at six sites. The small number of early neonatal deaths, compared with the much larger number of stillbirths, suggests that some early neonatal deaths may have been misclassified to conceal substandard care at birth, a relatively common phenomenon in low-resource settings [34]. Information was not available on how many CSs were performed on diagnosed intrauterine fetal deaths. The authors acknowledge the principle that to avoid greater risk to the mother, if the baby is already dead then it should be delivered vaginally where possible, while also bearing in mind that the specific characteristics of the case influences delivery. Nearly three-quarters of the files had no information on cause of perinatal death.

### Follow-up actions

Following the presentation of study results at each study site, stakeholders developed quality improvement action plans. Similar themes emerged, including the need to develop structured patient record forms; standardize CS indications; implement/improve partograph use, especially at referring centers; train and support staff in record-keeping practices; improve record room management; and streamline record-keeping systems. Since the completion of the study, partograph training has been implemented across all study sites. In Uganda, a coaching and mentoring program to improve partograph use has begun, where health personnel with partograph competencies help other providers develop skills through training and ongoing monitoring and feedback [35]. In Bangladesh, efforts are underway to conduct routine reviews of CS and to reinforce its appropriate use for valid clinical reasons (A.J. Faisel, personal communication, January 8, 2013).

### Methodological considerations

This study’s main limitation is the generalizability of results, as sites were not randomly selected. Thus, study sites may not be representative of similar sites in their respective countries. The data presented here are from a mix of private rural facilities and urban public hospitals. An advantage of data from a variety of facilities is that they highlight common deficits as well as priority areas for improving the quality of record keeping and care. A disadvantage of the heterogeneity of these data is that they are harder to interpret, hindering us from drawing firm conclusions about factors that contribute to service quality.

While retrospective record reviews are relatively less expensive to conduct than observational studies, this approach has limitations. The AMDD data collection tool that we adapted has been widely used as part of larger needs assessments for emergency obstetric services [15] in a variety of country settings, including Afghanistan [26], Angola, Ethiopia, Ghana, Guyana, and Malawi [P. Bailey, personal communication, January 13, 2013]; however, it has not been formally validated [26]. While data were missing on key variables, we were able to describe CS practices and identify areas needing improvement, such as partograph use and standardized terminology for CS indications. During chart reviews, one is obliged to accept at face value the information contained in the chart. However, it can be assumed that clinicians know what to write to make a procedure sound “medically justified”. Alternatively, appropriate care may have been provided but not recorded due to high work load. Ideally, data from chart reviews should be compared with observations of practices [26].

We did not collect detailed information about intrapartum care. Also, because we did not collect data about the availability of and capacity for providing emergency obstetric in each of the study site’s catchment areas, it is difficult to interpret institutional CS rates. Any future studies using this type of record review methodology to assess quality of care could be strengthened by including intrapartum care variables, as well as a review of all obstetric services available in the study site’s catchment area.

## Conclusions

This study highlights common shortcomings in CS record-keeping across a range of facilities in a variety of low-income countries. These include lack of documentation from referring facilities, inadequate use of the partograph, non-standardized terminology for CS indications, and poor documentation of the decision-to-delivery interval. While the volume of missing data and confusing non-standardized terminology found in the records limits our ability to draw conclusions about the quality of CS care at these sites, the poor maternal and fetal outcomes revealed by the data speak for themselves. Our study findings identified record-keeping deficits, which can serve as a practical guide to the essential elements of care that should be included in CS audit for quality improvement. In addition, these findings underline Graham and colleagues’ recent call for increased attention to and investment in medical record keeping as a vital, yet neglected, aspect of increasing the visibility of and accountability for women and babies within health systems [22]. The ability to retrace a woman’s path to CS is fundamental to identifying and addressing missed opportunities at critical junctures of care.

## Competing interests

The authors declare that they have no competing interests. The findings presented in the paper are part of larger study conducted by EngenderHealth’s Fistula Care project to assess the quality of CS. The study was funded by the United States Agency for International Development (USAID). Stated opinions are those of the authors and do not necessarily reflect the views of USAID or EngenderHealth.

## Authors’ contributions

EL and JR designed the study; EL served as primary investigator and coordinated the drafting and finalization of the manuscript. RF led the fieldwork in Niger and Guinea. JR and CP led the analysis and categorization of the indications into groups. RF, JR, CP, and CM contributed to the overall analysis and provided critical input and review to the manuscript. All authors read and approved the final manuscript.

## Authors’ information

EL (MPH) is former Deputy Director of Fistula Care *Plus* at EngenderHealth; CP (RN/RM, MPH) is former Medical Associate for Fistula Care at EngenderHealth; JR (MD) is Clinical Director for Fistula Care *Plus* at EngenderHealth; RF (MPH) is a student midwife at SUNY Downstate Medical Center, formerly Senior Monitoring and Evaluation and Research Associate at EngenderHealth; CM (M.Sc.) is a doctoral student at McMaster University, formerly graduate school intern at EngenderHealth.

## Pre-publication history

The pre-publication history for this paper can be accessed here:

http://www.biomedcentral.com/1471-2393/14/139/prepub

## Supplementary Material

Additional file 1 Fistula Care Record Review Data Collection Tool. Click here for file
