# Supplementary material for: Assessing the quality of record keeping for cesarean deliveries: results from a multicenter retrospective record review in five low-income countries
Source: BMC Pregnancy Childbirth. 2014 Apr 12;14:139. doi: 10.1186/1471-2393-14-139 (PMC4021100; doi:10.1186/1471-2393-14-139)
Supplement: Additional file 1 — Fistula Care Record Review Data Collection Tool. [file 1471-2393-14-139-S1.docx]

**Additional file 1. Fistula Care Record Review Data Collection Tool**

**CESAREAN DELIVERY RECORD REVIEW^^[[1]](#footnote-1)^^**

**Interviewer Name** _____________________________ **Date** (d/m/y): ___ / ___ / ___

**Name of Facility __________________________________________________________**

***Instructions:*** *Consult the birth registers and/or the operating room registers to identify Cesarean deliveries performed in the last calendar year. Each page contains columns for 5 women (you will need to use multiple pages). Follow the instructions provided about drawing the sample of records to review. Put the response code for each question in the appropriate column. Ask to see the clinical card and partograph to verify each aspect assessed. If the response to the question is not documented in the register, clinical record, or in the partograph, consider that the activity was not performed and use the code for no information (9 or 99****)***

| **No.** | **Question** | **Case ___** | **Case ___** | **Case ___** | **Case ___** | **Case ___** |
| --- | --- | --- | --- | --- | --- | --- |
| 1 | Age of the woman (99=no information) |  |  |  |  |  |
| 2 | Parity of the woman (99=no information) |  |  |  |  |  |
| 3 | Residence of the woman  1. Urban 2. Rural  9. Unknown |  |  |  |  |  |
| 4 | Was the woman referred from another health facility?  1. Yes 0. No>SKIP to Q8 |  |  |  |  |  |
| 5 | If a women was referred from another health facility (yes to 4), did she arrive with a completed partograph?   1. Yes 0. No.   88. Not applicable |  |  |  |  |  |
| 6 | Did patient arrive with referral notes?  1. Yes 0. No ->SKIP to Q8  8. Not applicable |  |  |  |  |  |
| 7. | If a woman arrived with referral notes, do the referral notes indicate that she had had uterotonic initiation or augmentation of labor?  1. Yes 0. No 9. No data  8. Not applicable |  |  |  |  |  |
| 8. | Was the time of admission recorded?  1. Yes 0. No SKIP to Q10  9. No information >SKIP to Q10 |  |  |  |  |  |
| 9. | Time of admission (record using 24-hour clock)  99=No information |  |  |  |  |  |
| 10. | Was time of first exam recorded?  1. Yes 0. No>SKIP to Q12  9. No information>SKIP tO Q12 |  |  |  |  |  |
| 11. | Time of examination (record using 24-hour clock)  99. No information |  |  |  |  |  |
| 12 | Was the time the decision was made to do a CS recorded?  1. Yes 0. No >SKIP to Q14  99. No information >SKIP to Q14 |  |  |  |  |  |
| 13. | Time the decision was made to do CS (record using 24-hour clock)  99. No information |  |  |  |  |  |
| 14 | Was the time recorded when the skin incision was made?  1. Yes 0. No>SKIP to Q16  99. No information>SKIP to Q16 |  |  |  |  |  |
| 15. | At what time was the skin incision made?  Record time using 24-hour clock.  99. No information |  |  |  |  |  |
| 16. | What was the primary indication for CS?  1. Obstructed labor  2. Deformed pelvis  3. Failed trial of labor  4. Major antepartum hemorrhage &  placenta previa (grade 3 or 4)  5. Uterine rupture  6. Malpresentation (including  transverse, oblique, brow)  7. Failure to progress in labor  8. Prolonged labor  9. Failed induction  10. Previous cesarean delivery  11. Uterine scar from other previous  surgery  12. Antepartum hemorrhage,  excluding those listed above, but  including abruption of placenta  13. Eclampsia/severe preeclampsia  14. Cord prolapse/presentation  15. Fetal distress  16. Severe intrauterine growth  retardation  17. Breech presentation  18. Cephalopelvic disproportion  (CPD)  19. Maternal medical disease (e.g.,  sickle cell anemia, HIV)  20. Psychosocial/maternal/family  request  21. Precious baby  22. Multiple gestation  23. Vesico-vaginal fistula postrepair  24. Vesico-vaginal fistula  25. Other (specify by writing in cell)  99. No information |  |  |  |  |  |
| 17. | What was the secondary indication for the cesarean? Use the response list in Q. 16 above.  99. no secondary indication recorded |  |  |  |  |  |
| 18. | Cesarean would be classified as:  1. Emergency 2. Elective  9. No information |  |  |  |  |  |
| 19. | Did this woman have a previous cesarean delivery?  1. Yes 0. No🡪 SKIP to Q21  9. No information SKIP to Q21 |  |  |  |  |  |
| 20. | If this woman had a previous c-section, how many previous c-sections?   1. One 2. Two or more   88. Not applicable |  |  |  |  |  |
| 21. | Type of anesthesia used:  1. General 2. Spinal/epidural  3. Other (specify)  9. No information |  |  |  |  |  |
| 22. | What type of clinician performed the surgery?   1. General surgeon 2. Obstetrician/gynecologist 3. General practitioner 4. Non-physician clinician (health officer/clinical officer) 5. Other (specify by writing in cell)   9. No information |  |  |  |  |  |
| 23. | Was a partograph used to monitor labor,  0. Cesarean was elective, therefore no partograph SKIP to Q26  1. Partograph used *(type of partograph not important)*  2. Partograph not used SKIP to Q26  9. No information SKIP to Q26 |  |  |  |  |  |
| 24. | Was the partograph completed correctly? (according to site/MOH partograph protocol or, if nonexistent, WHO protocol)   1. Yes, completed correctly 2. Partially completed   8. Not applicable (no partograph) |  |  |  |  |  |
| 25. | IF the partograph was used, was the action line reached or crossed?  1. Yes  0. No SKIP to Q26  88. Not applicable  99. No information SKIP to Q26 |  |  |  |  |  |
| 25a. | If the action line was crossed, by how much? |  |  |  |  |  |
| 26. | Was time of the birth of baby recorded?  1. Yes 0. No skip to Q28 |  |  |  |  |  |
| 27. | Record time of birth using 24 hr clock)  99. no information |  |  |  |  |  |
| 28. | What was the outcome for the baby/babies?  1. Alive 🡪 skip to Q32  2. Dead  3. One alive, one/or more dead (multiple births)  9. No information 🡪 SKIP to Q32 |  |  |  |  |  |
| 29. | Was the death a stillbirth?  1. Yes, fresh stillborn (FSB)  2. Yes, macerated death (MSB)  3. One stillbirth, one/or more early neonatal deaths (multiple births)  4. No, not stillbirth  8. Not applicable |  |  |  |  |  |
| 30. | Was the death early neonatal?   1. Yes, <24 hours after delivery 2. Yes, >24 hours and less than 7 days after delivery 3. No, more than 7 days after delivery   8. Not applicable  9. No information |  |  |  |  |  |
| 31. | If fetal/early neonatal death, primary cause of death:  1. Prematurity  2. Asphyxia and birth trauma  3. Infection/pneumonia  4. Congenital anomalies  5. Other (specify by writing in cell)  6. Unknown  8. Not applicable  9. No information |  |  |  |  |  |
| 32. | What was the maternal outcome?  1. Alive 🡪 SKIP to Q36  2. Dead  9. No information 🡪 SKIP to Q36 |  |  |  |  |  |
| 33. | What was the timing of the mother’s death?   1. Before delivery of baby 2. Within 24 hours after delivery 3. During cesarean surgery 4. 2nd to 7th day after delivery 5. 7th to 42nd day after delivery   8. Not applicable  9. No information |  |  |  |  |  |
| 34. | If maternal death, primary cause of death  *(please copy the primary cause of death as recorded in the patient card or other source of information)*  77. Cause of death unknown  88. Not applicable  99. No information |  |  |  |  |  |
| 35. | If maternal death, secondary cause of death  *(please copy the secondary cause of death as recorded in the patient card or other source of information)*  88. Not applicable  99. No secondary cause of death listed |  |  |  |  |  |
| 36. | Did the mother experience any complications before she was discharged?  1. Yes  0. No SKIP to Q37  9. No information SKIP to Q37 |  |  |  |  |  |
| 36a. | If the woman experienced complications, record complications. |  |  |  |  |  |
| 37. | Were prophylactic antibiotics administered?  1. Yes 0. No  9. No information |  |  |  |  |  |
| 38. | Was there an infected wound (from current cesarean)?  1. Yes 0. No  9. No information |  |  |  |  |  |
| 39. | Does the patient record file include a signed informed consent form for surgery?  1. Yes 0. No. |  |  |  |  |  |
| 40. | Was the woman given a permanent method of contraception?  1. Yes 0. No  9. No information |  |  |  |  |  |
| 41. | If the patient received a permanent method, is there a signed informed consent form in the file?  1. Yes 0. No  88. Not applicable |  |  |  |  |  |

| **Comments** |
| --- |
|  |

1. Adapted from Module 8 Cesarean delivery record review in Needs assessment of Emergency Obstetric and New Born Care. AMDD, 2009. [↑](#footnote-ref-1)
